# Brazilian Guidelines for Early Mobilization in Intensive Care Unit

**DOI:** 10.5935/0103-507X.20190084

**Published:** 2019

**Authors:** Esperidião Elias Aquim, Wanderley Marques Bernardo, Renata Ferreira Buzzini, Nara Selaimen Gaertner de Azeredo, Laura Severo da Cunha, Marta Cristina Pauleti Damasceno, Rafael Alexandre de Oliveira Deucher, Antonio Carlos Magalhães Duarte, Juliana Thiemy Librelato, Cesar Augusto Melo-Silva, Sergio Nogueira Nemer, Sabrina Donatti Ferreira da Silva, Cleber Verona

**Affiliations:** 1 Associação de Medicina Intensiva Brasileira - São Paulo (SP), Brasil.; 2 Associação Médica Brasileira - São Paulo (SP), Brasil.; 3 Universidade de São Paulo - São Paulo (SP), Brasil.

**Keywords:** Respiration, artificial, Critical care, Early ambulation, Exercise, Mobility, Patient safety, Intensive care units

## Abstract

Immobility can cause several complications, including skeletal muscle atrophy and weakness, that influence the recovery of critically ill patients. This effect can be mitigated by early mobilization. Six key questions guided this research: Is early mobilization safe? Which patients are candidates for early mobilization? What are the contraindications? What is the appropriate dose, and how should it be defined? What results are obtained? What are the prognostic indicators for the use of early mobilization? The objective of this guideline was to produce a document that would provide evidence-based recommendations and suggestions regarding the early mobilization of critically ill adult patients, with the aim of improving understanding of the topic and making a positive impact on patient care. This guideline was based on a systematic review of articles conducted using the PICO search strategy, as recommended by the Guidelines Project of the *Associação Médica Brasileira*. Randomized clinical trials, prognostic cohort studies, and systematic reviews with or without meta-analysis were selected, and the evidence was classified according to the Oxford Center for Evidence-based Medicine Levels of Evidence. For all the questions addressed, enough evidence was found to support safe and well-defined early mobilization, with prognostic indicators that support and recommend the technique. Early mobilization is associated with better functional outcomes and should be performed whenever indicated. Early mobilization is safe and should be the goal of the entire multidisciplinary team.

## INTRODUCTION

In the last decade, there has been an increase in evidence regarding the functional benefit of early physical therapy for critically ill patients starting in the first 48 hours after the institution of mechanical ventilation (MV); however, patient mobilization as a usual practice is still infrequent.^(1-4)^ In Brazil, it was recently observed that no more than 10% of critically ill patients are mobilized out of bed.^(5)^

The consequences of immobility due to prolonged hospitalization and immobility associated with old age, disease severity and admission type (acute/elective) can last for up to 5 years after hospital discharge.^(6,7)^ This, immobility represents a public health problem as it increases comorbidities and the mortality rate, increases patient complexity and is burdensome for families and for the health system.

Although the arsenal of evidence is sufficient, and professionals recognize the benefits of early mobilization, its application is perceived as challenging. Increased knowledge and application guidelines can help reduce the barriers to the widespread, facilitated and safe implementation of this practice; this effort is the main purpose of this document.^(8)^

The task force of the European Respiratory Society (ERS) and the European Society of Intensive Care Medicine (ESICM) suggests that a hierarchy of mobilization activities based on a sequence of intensity and the repetition of exercises be established in the intensive care unit (ICU) and suggests that such activities be started as early as possible.

The multidisciplinary team should be responsible for identifying indications and contraindications for early mobilization, but it is up to the physiotherapist to define the best intervention model and its intensity, frequency, continuity or interruption. Reducing the hospitalization time of these patients and returning them to functionality are the major goals of the multidisciplinary team.

## METHODOLOGY

**Figure 1:**
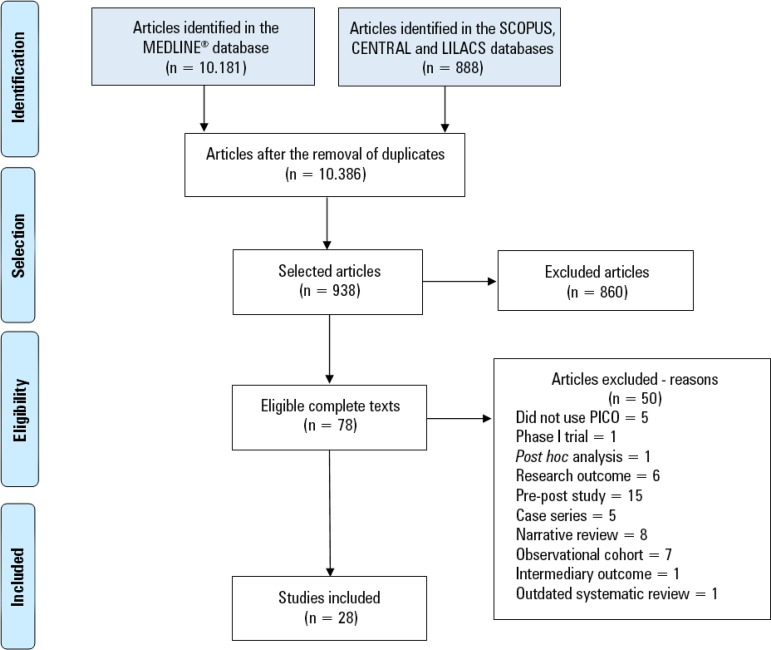
Design of the selected studies.

This guideline was developed based on a systematic review of articles using the PICO (Population, Intervention, Comparison, Outcome) search strategy, as recommended by the Guidelines Project of the *Associação Médica Brasileira*. The search strategy was based on the six clinical questions and was structured with Medical Subject Heading (MeSH) terms: adult patients ≥ 7 days hospitalized in the ICU and receiving MV, early mobilization, conventional treatment, length of hospital stay; length of stay in the ICU; duration of MV; mortality rate; hospital readmission 30 days after discharge; rate of return to work; level of mobility (extubation, discharge from the ICU, hospital discharge); functional status after discharge (at 30, 60, and 90 days); and adverse events. The studies were retrieved from the MEDLINE^®^, Scopus, LILACS, and Cochrane Central databases without date restriction, and study selection and the evaluation of the titles and abstracts were independently and blindly performed in strict accordance with the inclusion and exclusion criteria. Finally, studies with potential relevance were selected. When the title and the abstract were not illuminating, the article was read in its entirety. Only studies for which full texts were available and those that were written in Portuguese, English or Spanish were considered for critical evaluation. The designs of the selected studies were randomized clinical trials, prognostic cohort studies and systematic reviews with or without meta-analysis, and the evidence was classified according to the Oxford Center for Evidence-based Medicine Levels of Evidence. The degrees of recommendation were inserted next to the references. A total of 28 studies (16 randomized clinical trials, 3 systematic reviews, and 9 prognostic cohort studies) were selected to support the six guidelines (clinical questions) (Figure 1). The recommendations were prepared by the review authors with the initial characteristic of synthesis of the evidence and were submitted for validation by all the authors participating in the elaboration of the guideline. The overall synthesis was prepared considering the described evidence and its estimated power (Table 1).^(9)^

**Table 1:** Level of scientific evidence by study type, according to the Oxford Centre for Evidence-based Medicine in May 2001(9)

Degree of recommendation	Level of evidence	Treatment/Prevention - Etiology	Prognosis	Diagnosis	Differential diagnosis/prevalence of symptoms
A	1A	Systematic review (with homogeneity) of randomized controlled clinical trials	Systematic review (with homogeneity) of cohorts since the onset of the disease Prognostic criteria validated in several populations	Systematic review (with homogeneity) of diagnostic studies at level 1 Diagnostic criteria of level 1B studies in different clinical centers	Systematic review (with homogeneity) of a cohort study (contemporary or prospective)
	1B	Randomized controlled clinical trial with a narrow confidence interval	Cohort from the onset of disease, with a loss < 20% Prognostic criteria validated in a single population	Cohort validated with a good reference standard Diagnostic criteria tested in a single clinical center	Cohort study (contemporary or prospective) with few losses
	1C	Therapeutic results of the "all or nothing" type	Case series of the "all or nothing" type	Sensitivity and specificity close to 100%	Case series of the "all or nothing" type
B	2A	Statistical review (with homogeneity) of cohort studies	Systematic review (with homogeneity) of historical cohorts (retrospective) or follow-up of untreated cases from a randomized clinical trial control group.	Systematic review (with homogeneity) of diagnostic studies with a level > 2	Systematic review (with homogeneity) of studies on a differential diagnosis of level ≥ 2b
	2B	Cohort study (including randomized clinical trial of lower quality)	Historical cohort study Follow-up of untreated patients from a randomized clinical trial control group Prognostic criteria derived or validated only in fragmented samples	Exploratory cohort with a good reference standard Diagnostic criteria derived or validated from fragmented samples or databases	Historical cohort study (retrospective cohort) or study with a compromised follow-up (large number of losses)
	2C	Observation of therapeutic outcomes (research outcomes) Ecological study	Observation of clinical outcomes (research outcomes)		Ecological study
	3A	Systematic review (with homogeneity) of case-control studies		Systematic review (with homogeneity) of diagnostic studies with a level ≥ 3B	Systematic review (with homogeneity) of level ≥ 3B studies
	3B	Case-control study		Nonconsecutive selection of cases or reference standard applied in a rather inconsistent manner	Cohort with nonconsecutive case selection or a very limited study population
C	4	Case report (including cohort or case-control studies of lower quality)	Case series (and lower-quality prognostic cohort)	Case-control study or poor or nonindependent reference standard	Outdated case series or reference standard
D	5	Expert opinion without critical evaluation or based on basic materials (physiological study or animal study)			

## EXTRACTION OF RESULTS

### 1. Is early mobilization safe?

When discussing the safety of early mobilization, the main assumptions cited are adverse events, mortality (A)^(10-23)^ (B)^(24-28)^ and safety criteria.

Safety criteria should be assessed prior to initiation of mobilization for critically ill patients. Cardiovascular, respiratory and neurological parameters were the main parameters identified and described in the literature. From the cardiovascular point of view, the reference parameters are heart rate > 40bpm and < 130bpm, systolic blood pressure (SBP) > 90mmHg and < 180mmHg and mean arterial pressure > 60mmHg and < 110mmHg. From the respiratory point of view, the recommended safety criteria are respiratory rate > 5irpm and < 40irpm and peripheral oxygen saturation > 88%; if the patient is under MV, a fraction of inspired oxygen < 60% and/or a positive end-expiratory pressure (PEEP) < 10cmH_2_O is required. From the neurological point of view, the patient should not present increased intracranial pressure or be agitated; he or she must be able to properly understand and follow commands and open his or her eyes to verbal stimulation (B)^(29)^ (D).^(30)^

The main adverse events cited were cardiovascular effects; loss and/or displacement of endotracheal tubes; the interruption of early mobilization due to discomfort or fatigue, agitation, respiratory rate, pain or syncope; readmission due to polyarthralgia (most likely due to post-discharge interventions); decreased oxygen saturation; and patient-ventilator asynchrony. Even when adverse events occurred during early mobilization, they occurred with low frequency. In addition, adverse events can also occur regardless of early mobilization. They are considered nonserious, with the exception of reduced oxygen saturation, and do not require specific medical intervention or corrective treatment; only the discontinuation of mobilization is required. One of the most common reasons for discontinuing exercise during early mobilization was the refusal of patients or relatives when a protocol associated with cognitive therapy was used (A)^(10-23)^ (B).^(24-28)^

Pulmonary embolism, pneumothorax, severe cardiac arrhythmia, myocardial infarction and acute muscle injury were considered serious adverse events during the early mobilization in a protocol based on stages. Hypoxemia and tachycardia were also considered severe without the stages of rehabilitation. Pain in the acute dorsal region accompanied by hypertensive urgency occurred during early mobilization and was considered severe in a protocol associated with cognitive therapy, but it did not prevent participation in subsequent interventions. Adverse events can also be cited in terms of their cumulative incidence (frequency) without citing severity; frequent events included reduced oxygen saturation, hemodynamic changes and the removal or dysfunction of the intravascular catheter, in addition to tachypnea, bradycardia, patient intolerance, endotracheal tube loss and hypotension (A)^(10-23)^ (B).^(24-28)^

Mortality does not increase with early mobilization in either the hospitalization period or the post-discharge period (A),^(10-23)^ but it was identified as a significant protective factor (B)^(24-28)^ by the same ICU at 28 days and during the hospitalization period (A)^(10-23)^ (B).^(24-28)^

There was a significant mean difference in the duration of survival (days) during hospitalization and in the 6-months after discharge that favored the group that underwent early mobilization. The timing of early mobilization was also cited as a factor that promoted patient survival; the earlier the exposure to therapy, the higher the survival rate was (A)^(10-23)^ (B).^(24-28)^

The level of daily activity during interventions was related to the development of potential adverse events. However, adverse events were not related to exercise type or intensity. These events did not impact the need for additional therapy or increase the cost or length of hospital stay (A)^(10-23)^ (B).^(24-28)^

The major limitation of early mobilization was hemodynamic instability, and hypertension (SBP > 170mmHg) was considered a contraindication. The second limiting factor was related to respiratory dysfunction due to recent intubation/extubation, prone position or severe hypoxemia in patients with a specific fraction of inspired oxygen (FiO_2_) (A)^(10-23)^ (B).^(24-28)^ The application of ECMO can also be a limiting factor as it is independently associated with potentially serious adverse effects (A)^(10-23)^ (B).^(24-28)^

## RECOMMENDATION

Early mobilization is safe. Adverse events are mainly related to hemodynamic and/or respiratory changes, are low-frequency and are reversible with the interruption of the intervention. Adverse events are not frequent or severe, and early mobilization is considered safe (**A**).

### 2. Which patients are candidates for early mobilization?

The candidates for early mobilization are (A),^(10-23,31)^ (B):^(27,28,32-34)^

- Preferably, adults (age ≥ 18 years) hospitalized in the clinical or surgical ICU for at least 72 hours who are spontaneously breathing or who require 48 or more hours of invasive or noninvasive MV.
- Patients who are preferentially cooperative and without intracranial hypertension, with hemodynamic stability (defined as SBP > 90mmHg and < 170mmHg) and respiratory stability (preferably with oxygen saturation (SpO_2_) > 90%, FiO_2_ ≤ 60% and respiratory rate < 25irpm).

## RECOMMENDATION

Early mobilization is indicated for adults in the ICU, preferably those under spontaneous breathing, who cooperate and who do not have intracranial hypertension (**A**). Mechanical ventilation and noncooperation may be considered limitations for early mobilizations, but not contraindications.

### 3. What are the contraindications of early mobilization?

Early mobilization is contraindicated for patients with (A),^(11-20,23,31,35)^ (B):^(26,27)^

- Terminal diseases; systolic hypertension > 170mmHg; SpO_2_ < 90%, regardless of the FiO_2_; intracranial hypertension; unstable fractures; recent acute myocardial infarction; open abdominal wounds; and heart rate reduction of 20% or more during early mobilization activities. Deep cognitive and neurological deficits can be considered limitations but not contraindications.

## RECOMMENDATION

Early mobilization is contraindicated for terminal patients with systolic hypertension (systolic blood pressure > 170mmHg) or intracranial hypertension, unstable fractures, recent acute myocardial infarction and open abdominal wounds (**A**).

### 4. What is the appropriate dose of early mobilization, and how should it be defined?

Accurate clinical decision-making is fundamental to the effectiveness of physical therapy practices. Dosage should be based on clinical efficacy (A)^(10,11,13-19,31,35)^ (B),^(36)^ the individual tolerance of each patient, the patient's age and his or her previous condition.

An often-found care flow considers functionality in terms of the domains of locomotion and transfer, namely, starting from the lying position and progressing to the sitting position, then the standing position, and then to walking. The interventions tested ranged from positioning to more intense physical activity, and progression along the described flow is frequently associated with an increased dose (A)^(10,11,13-19,31,35)^ (B).^(36)^

4.1. Passive mobilization - Approximately 10 to 20 mobilizations per selected joint, up to two times/day. In cases of joint stiffness, passive mobilization may include accessory movements or slides, with the aim of increasing the range of motion. Measurement of the range of motion by goniometry can be performed periodically to evaluate the possible range gain.
4.2. Active exercises - One hour per day, up to two 30-minute sessions. Active exercises should include functional movements (usually on diagonals, combining, for example, flexion, adduction and external rotation of the upper limb with elbow flexion to bring the hand to the mouth for feeding) that can serve as a basis for activities of daily living. Active exercises should include not only the transfer from a lying to a sitting position but also weight transfers to the sides, forward and backward and trunk rotation in the sitting position to ensure this essential function is stable and can be performed safely and with adequate trunk control.
4.3. Positioning and progression - Assisted verticalization with an orthostatic board: up to 1 hour per day, up to twice a day; sitting in an armchair: up to 90 minutes, up to twice a day. The trunk control described in topic 4.2 should be addressed in combination and not only by keeping the patient in the sitting position. Orthostatic posture should be adopted with physiotherapeutic assistance. Balance should be addressed in this position, with weight transfer to both sides, forward and backward, along with the first steps test. Patients who are able to remain standing in a stable way should be encouraged to begin walking.
4.4.Ergometer cycling - The main objective of this activity is to improve cardiovascular conditioning. Monitoring of at least the heart rate, blood pressure and SpO_2_ should be provided. Passive ergometer cycling: 20 minutes, with 20 cycles per minute. Active: two 10-minute sessions per day, lasting up to 30 to 40 minutes.

## RECOMMENDATION

The appropriate dose of early mobilization is defined by clinical efficacy and individual tolerance (**B**).

The doses are as follows:

- Passive mobilization: approximately 10 to 20 mobilizations per selected joint, up to two times/day.
- Active exercises: 1 hour per day, up to two 30-minute sessions.

The following constitute positioning and progression:

- Assisted verticalization with an orthostatic board: up to 1 hour per day, up to twice a day.
- Passive ergometer cycling: 20 minutes, 20 cycles/minute.
- Active ergometer cycling: two 10-minute sessions per day (**A**).

### 5. What results are obtained with early mobilization?

5.1. Indications and contraindications - The care and safety criteria for early mobilization are simple and do not require specific monitoring. Hemodynamic and respiratory stability, as well as adequate nutritional and cardiac reserves for the proposed intervention model, are described in a safe intervention model. The incidence of adverse events during the proposed intervention did not show a level of evidence that would justify the non-performance of early mobilization (A),^(10,12-23,31,35)^ (B).^(25,36)^
  It is suggested that adequate monitoring be performed during the intervention and that the intervention should be interrupted without prejudice to the patient when any adverse effect occurs (A),^(10,12-23,31,35)^ (B).^(25,36)^
5.2. Intervention model - Successful implementation of an early mobilization project is associated with the involvement and knowledge of the multidisciplinary team in collaboration with the patient, caregivers and/or family members. The results depend on periodicity, intensity and, especially, well-established goals (A),^(10,12-23,31,35)^ (B).^(25,36)^
  Functional scales such as the Functional Status Score (FSS) and the ICU Mobility Scale should be used to assess the functional response to early mobilization. The choice of and indications for the best intervention model depend on the patient's previous functional condition and daily evaluations of the patient's progression, and the dose is based on the functional gains presented (A),^(10,12-23,31,35)^ (B).^(25,36)^
5.3. Length of stay in the ICU and in the hospital - The proposed early mobilization intervention does not significantly affect patients' length of ICU or hospital stay; however, the functional improvement of patients treated with this intervention model at the time of discharge from the ICU and hospital is evident (A),^(10,12-23,31,35)^ (B).^(25,36)^
  Precocity and the establishment of clear goals, with phases of progressive intervention through functional diagnosis, are relevant factors and are directly related to functional independence and walking at the time of discharge (A),^(10,12-23,31,35)^ (B).^(25,36)^
5.4. Mortality - Early mobilization performed according to well-established safety criteria does not promote or increase patient mortality.
  The adverse event of mortality may vary according to the patient's previous functional condition, age and comorbidities prior to or acquired in the ICU (A),^(10,12-23,31,35)^ (B).^(25,36)^
5.5. Postdischarge functionality - Greater functional independence, greater tolerance of physical activities and the development of activities of daily living are related to early mobilization. Progressive increases in overall muscle strength and improvement of the overall condition depend on multiprofessional follow-up, even after discharge (A),^(10,12-23,31,35)^ (B).^(25,36)^

## RECOMMENDATION

The care and safety criteria for early mobilization do not require specific monitoring, and hemodynamic and respiratory stability characterize a safe intervention model (**A**).

### 6. What are the prognostic indicators for the use of early mobilization?

Assessment of the risk of functional decline should be part of the approach to critically ill patients. Pre-existing factors such as advanced age (associated with limitations), functional disability, geriatric syndromes and psychiatric disorders are among the domains to be considered, both to establish the approach to intervention and to predict the functional prognosis (A).^(37)^

The prognostic components tested include non-modifiable prior conditions, such as age and functional condition, which have demonstrated a negative influence in cases of extreme age and disability, respectively (A)^(10,20)^ (B).^(26,27,31-34,38,39)^

The conditions present throughout hospitalization that are modifiable or relatively modifiable represent the greatest risk contingent. These are (A)^(10,20)^ (B).^(26,27,31-34,38,39)^

6.1. Weight - Lighter patients (average weight 78kg) with an absence of coma or delirium were more often mobilized outside the bed than those with an average weight greater than 92kg.
6.2. Mechanical ventilation - Patients without ventilatory support were more often mobilized than those undergoing invasive MV.
6.3. Functional range - Greater functional range was related to higher previous levels of function. The highest and most frequently achieved level of activity that patients achieved in the ICU was performed in the armchair (on average 2.5 days after admission to the ICU) without transferring to the standing position.
  Patients who walk in the ICU are younger patients and have a shorter hospitalization time than those who do not walk in the ICU. Patients who did not achieve mobility beyond the bed in the ICU showed a much greater delay when sitting and walking; those functional levels were achieved 5 and 7 days after admission to the ICU, respectively. These individuals also spent more time in the ICU and the hospital. There was, however, a weak but significant negative relationship between the first time of walking in the hospital and walking on the day of discharge. Less than half of the patients evaluated (41%) were able to walk on the day of discharge.
6.4. Muscle strength - According to the Medical Research Council (MRC) test, mobilization had a dose-response effect in which muscle strength recovery during the ICU stay was higher in patients who remained at the sitting level for longer.
6.5. Most commonly reported mobility-limiting factors - Hemodynamic instability, followed by respiratory dysfunction due to recent intubation and extubation, were the most commonly reported mobility-limiting factors.
6.6. Protective factor - Early mobilization was not associated with increased mortality; on the contrary, it was identified in all multivariate models as a significant protective factor for the reduction of ICU mortality at 28 days and intrahospital mortality.
6.7. Sedation - The daily interruption of sedation in nonneurological patients was part of the management of delirium and early mobilization. The patient's mobility level was assessed using the Surgical Optimal Mobility Score (SOMS) (from zero to 4) to quantify the patient's mobilization capacity at fixed times and not necessarily after awakening. Most patients (85%) were able to perform passive range-of-motion exercises in bed, either lying or sitting. Only 2% of cases exhibited the ability to walk.
6.8. Length of stay in the intensive care unit - Length of stay in the ICU was inversely associated with activity level: a one-point improvement on the SOMS decreased the length of stay in the ICU by 11.1% and was directly associated with the Simplified Acute Physiology Score II (SAPS II) and the comorbidity index.
6.9. Duration of mechanical ventilation - Logistic regression was used to examine a series of factors that predict a duration of MV > 7 days. Lower scores on the Glasgow Coma Scale and higher partial pressure of carbon dioxide (PaCO_2_) were significantly associated with a duration of MV ≥ 1 week. The risk of MV use for ≥ 7 days was lower in patients who received early mobilization.

## RECOMMENDATION

The prognostic indicators include an assessment of the risk of functional decline, weight, functional range, muscle strength, hemodynamic instability, respiratory dysfunction, recent extubation, protective factors, sedation, length of stay in the ICU and duration of mechanical ventilation (**B**).

## GENERAL RECOMMENDATIONS

Early mobilization is safe; adverse events are mainly related to hemodynamic and/or respiratory changes, and there are of low frequency and are reversible with interruption of the intervention (A). Early mobilization is safe and associated with a low incidence of adverse effects (B).

For patients who exhibit dyspnea during spontaneous breathing due to early mobilization, noninvasive ventilatory support must be provided to minimize respiratory discomfort. For patients undergoing invasive MV who experience respiratory discomfort and/or patient/ventilator asynchrony as a result of early mobilization, the ventilator should be adjusted to provide greater synchronicity (A).

The therapy employed should have effectiveness as a principle, i.e., social reintegration under conditions in which the impacts of hospitalization are minimized or reversed and the ability to perform activities that ensure independence in the community.

Independence in locomotion and transfer are therapeutic targets and should be actively pursued by the entire ICU multidisciplinary team.

Safety measures should be adopted by the multidisciplinary team so that if adverse effects occur, they are of minimal importance and are promptly resolved. Notification of adverse effects, as well as their resolution or mitigating factors, is of paramount importance so that they can be quickly resolved.

Early mobilization is associated with better functional results and should be provided whenever indicated, with respect for the contraindications, limitations and biological variations in adults.

Mobilization should be a paramount goal of the intensive care multidisciplinary team.

The prescription of activities and the developmental stages of the proposed tasks are the specific domain of the physical therapist.

The prognostic factors (modifiable and nonmodifiable) for the risk of functional decline allow an estimation of ICU patients' adherence or response to early mobilization.

Modifiable barriers must be addressed by the multidisciplinary team to make early mobilization possible.
